# Effects of diacylglycerol-enriched alpha-linolenic acid oil on skin properties in mild skin discomfort: a randomized, double-blind, placebo-controlled study

**DOI:** 10.1038/s41598-025-34887-3

**Published:** 2026-01-06

**Authors:** Satoko Fukagawa, Yoshie Shimotoyodome, Keimon Sayama, Aya Sasaki, Katsuyoshi Saito, Hiroki Fujita, Yuki Shimizu, Koichi Misawa, Shinichiro Saito, Junko Ishikawa, Noriyasu Ota, Takehiko Yokomizo, Masanobu Hibi

**Affiliations:** 1 Human Health Care Products Research, Kao Corporation, Bunka 2-1-3, Sumida, Tokyo 131-8501 Japan; 2 Department of Biochemistry, Juntendo University Graduate School of Medicine, Hongo 2-1-1, Bunkyo, Tokyo 113-8421 Japan; 3 Biological Science Research, Kao Corporation, Akabane 2606, Ichikai-Machi, Haga-Gun, Tochigi, 321-3497 Japan

**Keywords:** Alpha-linolenic acid, Diacylglycerol-enriched alpha-linolenic acid oil, IgE, Omega-3 polyunsaturated fatty acids, Skin surface lipid RNA, Disease prevention, Allergy

## Abstract

Skin barrier function impairment and inflammation promote allergen invasion through the skin, leading to sensitization and exacerbation of allergic diseases. Intake of linseed oil, an oil rich in alpha-linolenic acid (ALA), suppresses inflammation and allergic symptoms. To our knowledge, the effects of diacylglycerol-enriched ALA (ALA-DAG) oil intake on skin properties and allergic symptoms have not been evaluated. We performed a double-blind, randomized, placebo-controlled, parallel-group study of 60 individuals aged 20–59 years with mild skin discomfort, including dryness, itching, and redness, to investigate the effects of ALA-DAG intake on skin and allergic symptoms. Participants were divided into two groups treated with either 2.5 g/day of ALA-DAG or placebo oil for 8 weeks. The skin properties were measured at baseline and at 8 weeks. Allergic symptoms were measured at 4 and 8 weeks. Compared with placebo oil intake, ALA-DAG intake increased skin hydration in the cheek, reduced nasal congestion and nose itchiness, and decreased mite-specific immunoglobulin E in the blood. Subgroup analysis showed that participants who were positive for mite allergen sensitization had reduced facial redness after ingesting ALA-DAG. These findings suggest that ALA-DAG intake improves skin properties and alleviates allergic symptoms.

**Supplementary Information:**

The online version contains supplementary material available at 10.1038/s41598-025-34887-3.

## Introduction

The global prevalence of allergic conditions, including allergic rhinitis and allergic asthma, is increasing^1–3^. Dysfunction of protective skin surface barriers and the associated immune dysregulation play an important role in the pathogenesis of allergic disease. Skin barrier integrity is compromised by temperature fluctuations, reduced humidity, inadequate nutrition, and psychological stress^4–6^. Disruption of the skin barrier increases sensitivity to external irritants, triggering inflammatory responses that manifest as skin itchiness and redness. Skin barrier dysfunction may also promote allergen entry and trans-epidermal sensitization, i.e., production of allergen-specific immunoglobulin E (IgE) antibodies and the induction of type 2 inflammation. According to the “allergic march” concept, atopic dermatitis often precedes the onset of food allergies and may progress to other allergic conditions, such as allergic rhinitis and asthma^7–9^. Although atopic dermatitis is not classified as an IgE-mediated allergic disease, the Japanese Clinical Practice Guidelines for Atopic Dermatitis^10^ indicate a close association with IgE sensitization and identify it as one of the gateways to the allergic march. Most individuals who develop atopic dermatitis have an atopic diathesis characterized by a family history of asthma, allergic rhinitis, conjunctivitis, or atopic dermatitis. This is typically associated with elevated total IgE levels and/or the presence of allergen-specific IgE antibodies, which are often correlated^10^.

A high prevalence of allergen-specific IgE antibodies has been demonstrated in healthy Japanese adults^11^. Although characterizing individuals with an allergic predisposition is important toward preventing allergic disease, few studies have thoroughly investigated the associated skin properties and symptoms. Individuals with an allergic predisposition often experience uncomfortable skin symptoms such as dry, itchy skin and redness, especially during seasonal changes. Effectively preventing the development of atopic dermatitis and other allergic diseases in these individuals requires maintaining optimal skin health and regulating excessive inflammatory responses to external stimuli. In addition, relieving skin symptoms such as dryness, itching, and redness is essential for enhancing quality of life.

Some kinds of dietary oils can contribute to immune regulation and attenuate inflammation and allergic reactions^12,13^. Polyunsaturated fatty acids containing two or more cis-double bonds have various physiological effects. Linoleic acid, an ω6 fatty acid, is converted to arachidonic acid by fatty acid elongases and desaturases, and is subsequently metabolized into inflammatory and allergenic lipid mediators^14^. The ω3 fatty acid alpha-linolenic acid (ALA, 18:3n-3), on the other hand, is converted to eicosapentaenoic acid (EPA) and docosahexaenoic acid (DHA) in the mammalian body and further transformed into anti-inflammatory lipid mediators^15^. Dietary intake of linseed oil, which is rich in ALA, inhibits allergic diarrhea, contact dermatitis, allergic rhinitis, and allergic conjunctivitis in animal models^16–19^. Human clinical trials have demonstrated that linseed oil intake decreases skin sensitivity and improves skin properties^20,21^.

Diacylglycerols (DAG), glycerides in which two fatty acids are bound to a glycerol backbone, are present in many natural dietary oils at levels ranging from 0.8% in grapeseed oil to 9.5% in cottonseed oil^22^. Orally ingested DAG is not readily resynthesized into triacylglycerol after absorption in the intestinal tract because the lipases selectively hydrolyze DAG, producing mainly 1-monoacylglycerol, which has limited availability for triglyceride synthesis^23^. A recent study reported that long-term continuous intake of DAG-enriched ALA oil (ALA-DAG) reduced serum triglyceride levels^24^. In addition, compared with linseed oil consumption of ALA-DAG reduces visceral fat mass area and the body mass index^25^. These findings suggest that ALA-DAG intake facilitates the efficient delivery of ALA and its metabolites to various tissues, thereby enhancing its effects, including its anti-inflammatory actions. Therefore, it is expected that ALA-DAG intake will improve skin properties and suppress allergy symptoms, but these effects of ALA-DAG have not yet been investigated.

The present study investigated the effects of 8 weeks of daily ALA-DAG intake on skin properties and allergic symptoms in individuals with mild skin discomfort, such as dryness, itching, and redness.

## Results

### Participants

**Fig. 1:**
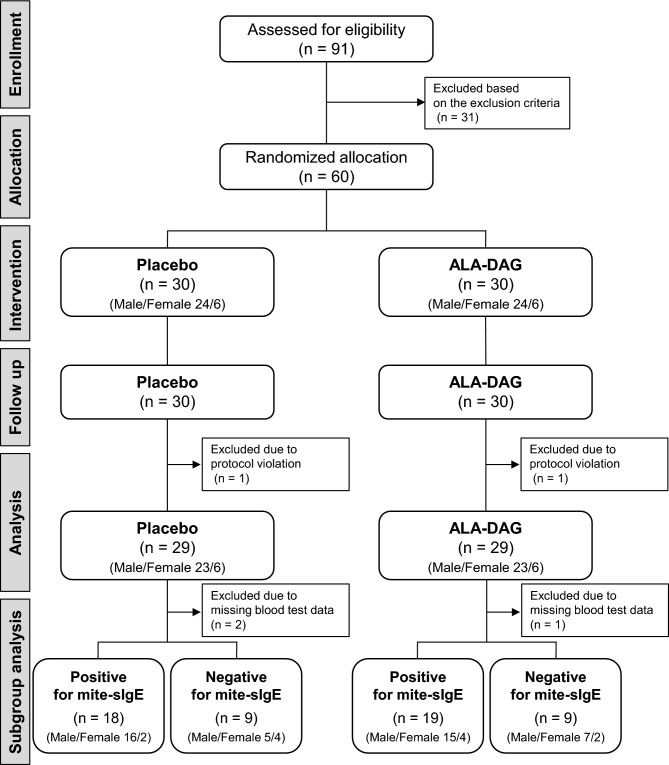
Test flow diagram of participants through each stage of the randomized double-blind, placebo-controlled study. *ALA-DAG* diacylglycerol-enriched alpha-linolenic acid oil, *sIgE* specific IgE.

A flow diagram from participant recruitment to analysis of the intervention study is shown in Fig. 1. Sixty eligible participants were assigned to the ALA-DAG group (n = 30, male/female = 24/6, age [means ± SD] 43.0 ± 6.1 years) or the placebo group (n = 30, male/female = 24/6, age 41.8 ± 9.0 years). All participants in both groups continued with their assigned intervention and completed the study. Intake of the test foods was greater than 80% for all participants. No adverse events occurred during the study that could be attributed to either intake of the test oils or measurement and evaluation. Of the 60 participants who completed the study, 58 were included in the study population (PPS, Per Protocol Set), excluding 2 participants who failed to adhere to the study protocol.

### Skin properties

Table 1 shows baseline values and changes from baseline in skin properties after 8 weeks. After the 8-week intervention, the change in skin conductance was significantly greater in the ALA-DAG group than in the placebo group (*P* = 0.014, Table 1). No differences in transepidermal water loss (TEWL) or skin color were detected between groups.

**Table 1:** Baseline values and changes from baseline after 8 weeks in skin properties and serum IgE levels compared between groups.

	ALA−DAG	Placebo	p−value
TEWL (g/m^2^/h)			
	N = 29	N = 29	
Baseline	37 (9)	37 (8)	
Change from baseline	−5 (8)	−6 (7)	0.441
Conductance (μS)			
	N = 29	N = 29	
Baseline	163 (79)	175 (127)	
Change from baseline	46 (69)	5 (61)	0.014
Brightness (L*, arb.unit)			
	N = 29	N = 29	
Baseline	53.0 (3.6)	54.1 (3.1)	
Change from baseline	0.31 (1.24)	0.28 (0.94)	0.914
Redness (a*, arb.unit)			
	N = 29	N = 29	
Baseline	13.04 (1.06)	12.65 (1.42)	
Change from baseline	−0.63 (0.66)	−0.39 (0.85)	0.272
Yellowness (b*, arb.unit)			
	N = 29	N = 29	
Baseline	19.54 (1.41)	19.65 (1.65)	
Change from baseline	0.63 (0.80)	0.70 (1.38)	0.769
Total IgE (IU/mL)			
	N = 28	N = 27	
Baseline	254.82 (271.66)	299.22 (344.74)	
Change from baseline	15.00 (58.53)	96.44 (476.65)	0.630
Mite−specific IgE (U_A_/mL)			
	N = 20	N = 20	
Baseline	16.40 (18.57)	21.05 (24.98)	
Change from baseline	−0.05 (4.29)	2.24 (4.42)	0.030
Japanese cedar pollen−specific IgE (U_A_/mL)			
	N = 25	N = 23	
Baseline	7.50 (9.44)	13.47 (15.44)	
Change from baseline	3.27 (4.49)	6.08 (7.39)	0.401

Numbers are expressed as Mean (SD). Wilcoxon rank-sum exact test was used for the comparison between groups.

### Total and allergen-specific IgE antibodies

**Fig. 2:**
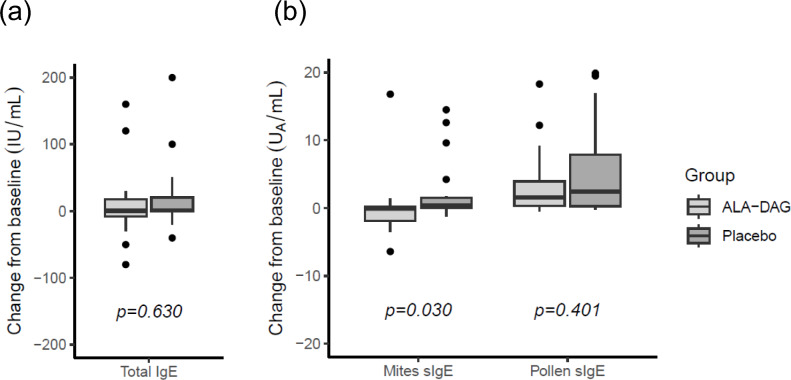
Changes from baseline in serum IgE antibodies after the 8-week intervention. (**a**) Total IgE. (**b**) Mite-specific IgE and Japanese cedar pollen-specific IgE. Between group comparisons were performed using the Wilcoxon rank-sum exact test. One outlier in the placebo group (total IgE > 200) was excluded from the analysis in panel (**a**). Sample sizes (placebo/ALA-DAG): n = 27/28 for (**a**), n = 20/20 (mite-specific IgE) and n = 23/25 (Japanese cedar pollen-specific IgE) for (**b**). *ALA-DAG* diacylglycerol-enriched alpha-linolenic acid oil, *sIgE* specific IgE.

Baseline values and changes from baseline in total IgE, mite-specific IgE, and Japanese cedar pollen-specific IgE antibodies in the serum after the 8-week intervention are shown in Table 1 and Fig. 2. At baseline, total IgE levels did not differ significantly between groups. Prior to the intervention, 19 ALA-DAG group participants and 18 placebo group participants were positive for mite‑specific IgE antibodies, and 22 and 24 participants, respectively, were positive for Japanese cedar pollen‑specific IgE antibodies, with comparable numbers between groups. The change in mite-specific IgE levels was significantly lower in the ALA-DAG group compared with the placebo group after the 8-week intervention (*P* = 0.030, Fig. 2b). The change in total IgE and cedar pollen-specific IgE during the 8-week study period did not differ significantly between groups.

### Fatty acids in the blood

Compared with the baseline values, plasma ALA levels were significantly increased in the ALA-DAG group after the 8-week intervention (Supplementary table 1). Over the 8-week study period, blood levels of palmitic acid increased significantly in both the placebo and ALA-DAG groups, whereas blood levels of dihomo-gamma-linolenic acid decreased significantly in both the placebo and ALA-DAG groups. No significant differences in the other fatty acids assessed were detected between groups. The change in the blood ALA levels negatively correlated with the change in arachidonic acid before and after the 8-week intervention in both the placebo (R = −0.386, *P* = 0.039) and ALA-DAG (R = −0.628, *P* < 0.001) groups (Supplementary Fig. 1).

### Quality-of-life questionnaires for skin conditions

Table 2 shows the comparison of the Skindex-16 scores between groups. The skin itchiness score after 4 weeks was significantly lower in the ALA-DAG group than in the placebo group (*P* = 0.044, Table 2), with a trend toward a group-by-time interaction (*P* = 0.060).

**Table 2:** Comparison of Skindex-16 scores between groups.

Items	Groups	Week 4		Week 8		RM-ANOVA; p-value	
		LS-Mean (SE)	p-value	LS-Mean (SE)	p-value	Group effect	Group x time
Symptoms	ALA-DAG	20.208 (3.036)	0.834	20.208 (2.948)	0.853	0.993	0.701
	Placebo	21.027 (2.437)		19.447 (2.861)			
Itching on the skin	ALA-DAG	30.116 (3.642)	0.044	34.139 (3.813)	0.817	0.197	0.060
	Placebo	41.148 (4.118)		35.401 (3.908)			
Burning or stinging sensation on the skin	ALA-DAG	18.597 (3.831)	0.482	18.023 (3.650)	0.853	0.606	0.591
	Placebo	14.736 (3.876)		17.035 (3.832)			
Pain in the skin	ALA-DAG	15.828 (3.683)	0.226	13.529 (2.903)	0.153	0.090	1.000
	Placebo	10.609 (2.321)		8.310 (2.231)			
Irritation sensation on the skin	ALA-DAG	16.224 (4.411)	0.777	15.075 (3.966)	0.688	0.679	0.920
	Placebo	17.684 (2.686)		17.109 (3.151)			
Emotions	ALA-DAG	20.903 (3.113)	0.706	24.844 (3.547)	0.199	0.631	0.076
	Placebo	22.823 (3.958)		18.742 (3.041)			
Functioning	ALA-DAG	6.359 (1.387)	0.844	6.244 (1.133)	0.309	0.592	0.249
	Placebo	5.940 (1.619)		8.698 (2.121)			

*LS-Mean* least squared mean, *RM-ANOVA* repeated measures analysis of variance, *Group x time* interactions of group and time. Generalized estimating equations were used for statistical modeling.

### Quality-of-life questionnaires for allergic rhinitis

Table 3 shows the between-group comparisons of the Japanese quality of life questionnaire for allergic rhinitis (JRQLQ) scores. Of the four nasal symptom parameters, the runny nose score showed a trend toward a group effect in repeated-measures analysis of variance (*P* = 0.082). The sneezing score at 8 weeks was significantly lower in the ALA-DAG group than in the placebo group (*P* = 0.021), indicating a significant group-by-time interaction (*P* = 0.009). The nasal congestion scores after 4 and 8 weeks were significantly lower in the ALA-DAG group than in the placebo group (*P* = 0.039 and *P* = 0.040, respectively), indicating a significant group effect (*P* = 0.017). The itchy nose score after 4 weeks was significantly lower in the ALA-DAG group than in the placebo group (*P* = 0.013), indicating a significant group effect (*P* = 0.014). In addition, the itchy eyes score after 8 weeks tended to be lower in the ALA-DAG group than in the placebo group (*P* = 0.092), with a trend toward a group effect (*P* = 0.096). The teary eyes score after 4 weeks was significantly lower in the ALA-DAG group than in the placebo group (*P* = 0.037), with a trend toward a group-by-time interaction (*P* = 0.079).

**Table 3:** Comparison of JRQLQ scores between groups.

Items		Week 4		Week 8		RM-ANOVA; p-value	
	Groups	LS-Mean (SE)	p-value	LS-Mean (SE)	p-value	Group effect	Group x time
Runny nose	ALA-DAG	0.807 (0.122)	0.140	0.879 (0.115)	0.111	0.082	0.989
	Placebo	1.117 (0.168)		1.186 (0.151)			
Sneezing	ALA-DAG	0.932 (0.122)	0.983	0.860 (0.101)	0.021	0.201	0.009
	Placebo	0.928 (0.134)		1.273 (0.145)			
Nasal congestion	ALA-DAG	0.674 (0.091)	0.039	0.674 (0.110)	0.040	0.017	0.658
	Placebo	0.970 (0.108)		1.039 (0.137)			
Itchy nose	ALA-DAG	0.225 (0.066)	0.013	0.547 (0.116)	0.131	0.014	0.506
	Placebo	0.668 (0.161)		0.846 (0.158)			
Itchy eyes	ALA-DAG	0.709 (0.120)	0.201	1.031 (0.126)	0.092	0.096	0.773
	Placebo	1.005 (0.197)		1.384 (0.169)			
Teary eyes	ALA-DAG	0.144 (0.050)	0.037	0.573 (0.117)	0.821	0.241	0.079
	Placebo	0.481 (0.146)		0.619 (0.156)			

*LS-Mean* least squared mean, *RM-ANOVA* repeated measures analysis of variance, *Group x time* interaction of group and time. Generalized estimating equations were used for statistical modeling.

### Subgroup analysis for mite-specific IgE levels

An exploratory, post‑hoc subgroup analysis in mite‑sensitized participants was conducted. The participants were divided into two groups based on their pre-intervention blood mite-specific IgE levels: IgE-positive [mite-specific IgE(+); ≥ 0.35 U_A_/mL] and IgE-negative [mite-specific IgE(-); < 0.35 U_A_/mL; Fig. 1]. In the placebo group, after excluding the 2 participants with missing blood test data, 18 participants (male/female = 16/2, age 41.8 ± 9.8 years) were mite-specific IgE(+) and 9 participants (male/female = 5/4, age 42.4 ± 7.6 years) were mite-specific IgE(-) before the intervention. In the ALA-DAG group, after excluding 1 participant with missing blood test data, 19 participants (male/female = 15/4, age 43.2 ± 6.0 years) were mite-specific IgE(+) and 9 participants (male/female = 7/2, age 43.3 ± 6.0 years) were mite-specific IgE(-) before the intervention.

Skin properties before the intervention were compared between the 2 groups: 37 participants (male/female = 31/6, age 42.5 ± 8.1 years) who were mite-specific IgE(+) and 18 participants (male/female = 12/6, age 42.1 ± 7.3 years) who were mite-specific IgE(-). The TEWL of the cheek was significantly higher in the mite-specific IgE(+) group (39.4 ± 8.6 g/m^2^/h) than in the mite-specific IgE(-) group (33.9 ± 6.2 g/m^2^/h; *P* = 0.039). No significant differences in skin conductance of the cheek or facial redness (a*), however, were detected between groups.

The effects of ALA-DAG were examined in the mite-specific IgE(+) and mite-specific IgE(-) groups. Analysis of 18 participants from the placebo group and 19 participants from the ALA-DAG group who were mite-specific IgE(+) before the intervention revealed that, compared with the placebo group, the ALA-DAG group showed significantly increased skin conductance, significantly reduced skin redness (a*), and significantly attenuated increases in mite‑specific IgE from baseline to week 8 (*P* = 0.042, *P* = 0.019, and* P* = 0.024, respectively). On the other hand, between the 9 placebo group participants and 9 ALA-DAG group participants who were mite-specific IgE(-) before the intervention, no significant differences in TEWL, conductance, or facial redness were observed (data not shown).

### Skin surface lipid gene expression

**Fig. 3:**
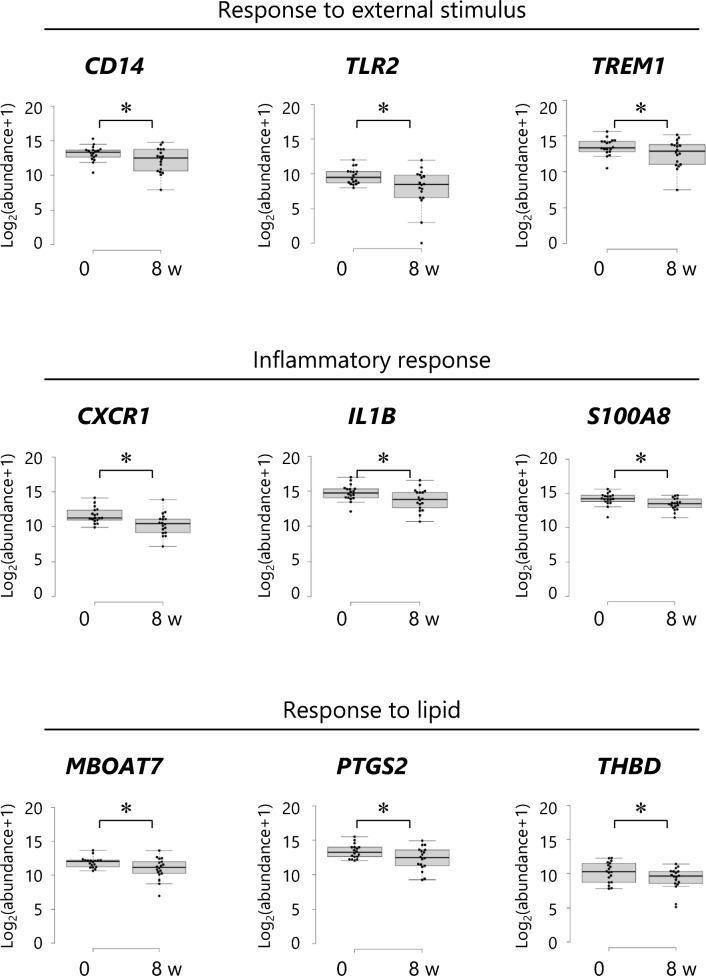
Downregulated genes in the skin surface lipids in the mite-specific IgE(+) ALA-DAG group (n = 18) from baseline (week 0) to week 8. * *P* < 0.05 vs baseline (Benjamini and Hochberg’s FDR < 0.05). *CD14* CD14 molecule, *CXCR1* C-X-C motif chemokine receptor 1, *FDR* false discovery rate, *IL1B* interleukin-1 beta, *MBOAT7* lysophospholipid acyltransferase 7, *PTGS2* prostaglandin-endoperoxide synthase 2, *S100A8* S100 calcium binding protein A8, *TLR2* Toll-like receptor-2, *THBD* thrombomodulin, *TREM1* triggering receptor expressed on myeloid cells 1.

Gene expression in skin surface lipids (SSL) was investigated to analyze the biomolecular changes associated with dietary ALA-DAG intake. Based on the subgroup analysis results, participants who were mite-specific IgE(+) at baseline were included. The SSL samples of 15 participants from the placebo group and 18 participants from the ALA-DAG group met the data analysis criteria (target detected ≥ 20%). In the differential expression analysis of SSL mRNA in the placebo and ALA-DAG groups before the intervention, 6437 genes meeting the quality criteria were identified, and no differentially expressed genes (DEGs) meeting the false discovery rate (FDR) threshold of < 0.05 were extracted. In the differential expression analysis before and after the intervention in the placebo group, 6828 genes were obtained and no DEGs were extracted (Supplementary Fig. 2a). On the other hand, differential expression analysis in the ALA-DAG group before and after the intervention identified 5892 DEGs, with 54 genes significantly downregulated after the intervention (FDR < 0.05, Supplementary Fig. 2b). Expression levels of the main genes identified are shown in Fig. 3. Gene ontology analysis of these 54 downregulated genes revealed significant enrichment of biological processes related to inflammation, response to external stimuli, and lipid response (FDR < 0.05, Supplementary Table 2).

## Discussion

In this double-blind, randomized, placebo-controlled, parallel-group study, the effects of 8-week daily intake of ALA-DAG on skin properties, skin discomfort, allergen-specific IgE, and subjective allergy symptoms were investigated in men and women experiencing mild skin discomfort, including dryness, itching, redness, and other allergic-like symptoms. Our findings revealed that, compared with placebo oil, continuous intake of ALA-DAG significantly increased skin conductance in the cheek and significantly decreased the skin itch score among various skin discomfort symptoms evaluated by the Skindex-16. Furthermore, intake of ALA-DAG suppressed the increase in mite-specific IgE in the blood and reduced subjective symptoms of eye and nose allergy as assessed by the JRQLQ. These novel findings suggest that continuous intake of ALA-DAG (2.5 g/day) improves skin function, reduces skin discomfort, and reduces allergy-related eye and nose symptoms.

For individuals with an allergic predisposition, it is essential to maintain healthy skin function and suppress excessive inflammatory reactions triggered by external stimuli. This approach can help prevent the development of allergic diseases such as atopic dermatitis and alleviate uncomfortable skin symptoms, ultimately improving quality of life. Linseed oil containing ALA, which has anti-inflammatory effects, is reported to improve skin properties^20,21^. In addition, increasing ω3 fatty acid intake reportedly decreases the incidence of allergies and other immune disorders because ω3 and ω6 fatty acids compete for shared metabolic pathways^26–28^. The effects of ALA-DAG on skin barrier function and allergy-related factors, however, have remained unclear. In the present study, skin barrier function, as measured by TEWL, improved with continuous ALA-DAG intake but was similarly improved by placebo oil intake. TEWL, a widely used skin property measure, fluctuates seasonally and is highest in winter when ambient temperature and humidity are low^29,30^. In our study, conducted from January to early April, the TEWL decreased even with the intake of placebo oil due to an increase in the outdoor temperature and humidity; thus, the effect of ALA-DAG for improving skin properties compared to the placebo oil might have been less detectable due to the greater influence of seasonal variation. On the other hand, continuous intake of ALA-DAG significantly increased the stratum corneum water content and significantly decreased the skin itch score among skin discomfort symptoms as assessed by the Skindex-16. Although the causal relationship between these two effects has not been directly determined, the reduction in itching observed after 4 weeks of ALA-DAG intake indicates that the inflammation-suppressing effect may have prevented the breakdown of the skin barrier caused by scratching, thereby maintaining skin moisture levels. These findings suggest that continuous intake of ALA-DAG may improve skin hydration and reduce skin discomfort.

Our results demonstrated that, compared with the placebo oil, daily intake of ALA-DAG for 8 weeks decreased subjective symptoms of eye and nose allergies in several categories, including runny nose, sneezing, nasal congestion, itchy nose, itchy eyes, and teary eyes. Although linseed oil intake is reported to effectively suppress allergic rhinitis and conjunctivitis in animal models^18,19^, no previous studies have evaluated the effects of ALA-DAG intake in humans. We found that mite-specific IgE levels, an indicator of allergy, increased moderately in the placebo group, but were suppressed in the ALA-DAG group. These results suggest that ALA-DAG intake alleviates allergy-related symptoms by influencing IgE production. In atopic dermatitis, impaired skin barrier function triggers an excessive immune response to external stimuli, such as allergens. This response leads to skin inflammation and itching, mediated by cytokines and mediators through the production of allergen-specific IgE. In individuals with atopic dermatitis, TEWL, a measure of skin barrier function, is increased^31–33^, and approximately 7–80% of individuals are reported to be positive for mite sensitization [mite-specific IgE(+)]^34–36^. On the other hand, the relationship between skin barrier function and mite sensitization in healthy subjects has not been fully investigated. The pre-intervention skin characterization of the study participants suggested that the TEWL of the cheek was significantly higher in the mite-specific IgE(+) group than in the mite-specific IgE(-) group, indicating that the skin barrier function was disrupted in the mite-specific IgE(+) group. These findings suggest that skin barrier function may be impaired in individuals predisposed to allergies, even if they have not been diagnosed with an allergic disease, and that this vulnerability may promote further allergen sensitization, creating a negative feedback loop. Furthermore, subgroup analysis of the efficacy of ALA-DAG in the group of mite-specific IgE(+) individuals with impaired skin barrier function indicated that ALA-DAG suppressed skin redness and an increase in mite-specific IgE. These results suggest that continuous intake of ALA-DAG effectively suppresses further increases in blood mite-specific IgE levels and reduces skin redness in individuals who are predisposed to allergies, such as those who test positive for mite sensitization, a skin condition suggested to be more sensitive to external stimuli. A survey of healthy Japanese adults reported a positive mite-sensitization rate of approximately 40–50%^11,37^, and ALA-DAG is expected to be particularly effective for reducing skin redness in these individuals. Cedar pollen-specific IgE was elevated in both the placebo and ALA-DAG intake groups in this study. Pollens generally induce sensitization through the nasal mucosa and airways. This study was conducted from January to early April in Japan, a period of high cedar pollen dispersion, and we speculate that ALA-DAG intake may not have been sufficient to suppress the cedar pollen-specific IgE elevation through the nasal and airway passages due to excessive exposure to pollen allergens. On the other hand, the importance of epicutaneous sensitization of mites has been reported^38^. The results of this study, in which mite allergen-specific IgE was suppressed by ALA-DAG intake, suggest that ALA-DAG contributes to reducing the effects of epicutaneous sensitization. We cannot exclude the possibility that differences in environmental allergen exposure between groups contributed to the smaller increase in mite‑specific IgE in the ALA‑DAG group. Environmental/household allergen levels were not measured in this study, however, so we cannot draw conclusions regarding exposure effects.

Analysis of SSL mRNA expression to investigate biomolecular changes revealed that ALA-DAG intake reduced the expression of genes related to inflammation, response to external stimuli, and lipid metabolism. SSL mRNA expression analysis, which can provide gene expression data for approximately 10,000 species from noninvasively collected sebum, has been used to study atopic dermatitis and sensitive skin. This method is considered one of the most effective tools for understanding the molecular pathogenesis of skin conditions due to its noninvasive specimen collection method^39–41^. A previous analysis of SSL mRNA in participants with atopic dermatitis revealed the upregulation of genes related to inflammation and immune responses^40,41^. In the present study, ALA-DAG decreased the expression of several inflammation- and immune response-related genes, including Toll-like receptor 2, interleukin 1B, and S100 calcium-binding protein A8, which are upregulated in the pathogenesis of atopic dermatitis^40,41^. The reduced skin redness and decreased expression of inflammation-related genes observed in the present study indicate that intake of ALA-DAG may suppress skin inflammation. In addition, ALA-DAG intake decreased the expression of prostaglandin G/H synthase 2 and lysophospholipid acyltransferase 7, which are related to lipid responses. These genes encode enzymes involved, respectively, in the synthesis of prostaglandins from arachidonic acid^42,43^ and in the conversion of arachidonic acid to phosphatidylinositol^44,45^. The gene expression changes observed in the present study may reflect some effects of ALA-DAG intake on the arachidonic acid cascade. As mentioned above, the ω3 fatty acid ALA competes with the metabolic pathway that converts the ω6 fatty acid linoleic acid to arachidonic acid, thereby inhibiting the production and activity of prostaglandins and other inflammatory eicosanoids. A negative correlation between changes in blood ALA and changes in arachidonic acid from pre‑ to post‑intervention was observed in both groups, but the correlation was stronger in the ALA‑DAG group. Considering that blood ALA levels increased only following ALA‑DAG intake, these findings suggest that the anti‑inflammatory effects of ALA‑DAG may be mediated, at least in part, by inhibiting the arachidonic acid cascade. The safety of ALA-DAG was discussed in a previous report^25^. In the present study, no adverse events were attributed to the test oil intake during the study period, suggesting that ALA-DAG is a safe and highly effective food.

In this study, individuals with subjective symptoms of skin dryness, itchiness, and redness who ingested ALA-DAG oil daily for 8 weeks showed a significant increase in skin hydration of the cheeks, a significant decrease in skin itchiness scores, and an improvement in subjective symptoms of eye and nose allergies compared to individuals ingesting the placebo oil. These findings indicate that ALA-DAG may be a safe and effective food component for maintaining healthy skin in individuals with dry, itchy, and red skin, and for alleviating the discomfort caused by allergy-like symptoms of the skin, eyes, and nose.

### Limitations

This study has several limitations. (1) Because this was an exploratory study, formal adjustments for multiple testing were not applied. Accordingly, the reported P-values are unadjusted and the results should be interpreted cautiously. The findings should be considered hypothesis-generating and require confirmation in adequately-powered, pre-specified trials with appropriate multiple testing management. (2) Randomization was stratified by age and sex, not by IgE status. To avoid underpowered interaction testing and potential inflation of type I error, we did not conduct additional subgroup analyses based on age or sex. (3) An exploratory subgroup analysis showed improvement in skin redness after ALA-DAG ingestion in mite-specific IgE(+) individuals, but additional studies involving a larger cohort of individuals with high mite-specific IgE levels are needed to validate these findings and establish their clinical significance. (4) Participants under treatment for atopic dermatitis or other allergic diseases were excluded per protocol, although a strong atopic background in some individuals is plausible and may contribute to variability in total and specific IgE. We could not add a post‑hoc endpoint such as the specific/total IgE ratio. Future studies should evaluate IgE normalization strategies (e.g., specific/total ratios) in larger confirmatory studies. (5) Because allergic symptom outcomes relied solely on participants’ subjective evaluations, future assessments should incorporate clinical evaluations of eye and nose allergy symptom scores. More comprehensive outcome assessment is needed to confirm the product’s clinical effectiveness in managing allergic symptoms.

## Materials and methods

### Participants

Participants were recruited through a website between December 2021 and January 2022. Selection criteria were (1) men and women between the ages of 20 and 59, (2) those with subjective symptoms of mild skin discomfort, such as dryness, itching, or redness, and (3) those who self-reported experiencing occasional allergy-like symptoms (e.g., sneezing, nasal congestion) without a clinical diagnosis of allergic disease. Exclusion conditions were as follows: (1) undergoing or scheduled to undergo treatment for skin and allergic diseases (atopic dermatitis, food allergy, bronchial asthma, allergic rhinitis or hay fever, conjunctivitis, etc.), (2) body mass index < 18.5 kg/m^2^ or ≥ 30.0 kg/m^2^, (3) experience of allergic symptoms due to the intake of linseed oil, perilla oil, or other edible oils in the past, (4) hospitalized within the last month due to illnesses that may affect this study (skin diseases, gastrointestinal, circulatory, or blood disorders, viral infections, etc.), (5) hepatitis B or C virus, syphilis virus, or HIV infection, (6) use of drugs that may affect the study, (7) regular consumption of linseed oil, perilla oil, or DHA/EPA supplements, (8) daily alcohol intake > 60 g/day on average, (9) smoking habit, and (10) pregnant or lactating. This study was performed in accordance with Ethical Guidelines for Medical and Health Research Involving Human Subjects. The study was approved by the Kao Corporation Ethics Committee for Human Trial Research (Approval No. K0047-2110, approved November 24, 2021) and registered with the University Hospital Medical Information Network (UMIN; http://www.umin.ac.jp/; Registration No. UMIN000046466, first registered 24/12/2021). Informed consent was obtained from all the participants.

### Study design

A double-blind, randomized, placebo-controlled, parallel-group study of 8 weeks’ daily intake of placebo or ALA-DAG oils from January to April 2022 was conducted to evaluate the effects of ALA-DAG intake on skin properties and allergic symptoms during the winter and spring when seasonal environmental changes are significant. The study was conducted in Tokyo, Japan. Screening and baseline measurements were performed on study applicants who lived in the vicinity of the study site, and eligible participants were selected based on the selection and exclusion criteria. Participants were stratified randomization with sex and age by study personnel not involved in the study. Subsequently, allocation to the ALA-DAG and placebo groups was performed by a different person. Participants consumed the designated test oils daily for 8 weeks and arrived at the study site for skin and allergy assessments at the end of the 8-week intake period. During the intervention period, participants were instructed to maintain their usual dietary habits but to avoid consuming any linseed oil, sesame oil, or DHA/EPA supplements, with no changes in other regularly used foods and supplements. No restrictions were imposed on the use of cosmetics or skincare products; however, participants were advised not to change their habitual use of such products throughout the study period. Alcohol was prohibited on the day before each test day, food was consumed at least 12 h before the test, and no food or drink other than water was allowed until completion of the test on testing day. Treatment allocation was concealed throughout the study (from screening to finalizing the data set) among all parties involved, including subjects, test food preparers, those in charge of allocation, and outcome assessors.

### Test foods

Participants consumed either manufactured linseed oil containing ALA-DAG (test oil; 2.5 g per day, constituting 0.9 g of alpha-linolenic acid esterified to DAG as fatty acid) or sunflower oil containing high oleic acid, an ω9 fatty acid (placebo; 2.5 g per day). The glycerides and fatty acid compositions of the test and placebo oils are shown expressed as weight percentages. For glycerides, the placebo oil predominantly contained triacylglycerols (TAG) at 97.9%, 2.1% DAG, and 0.0% monoacylglycerols (MAG) or free fatty acids (FFA). In contrast, the ALA-DAG oil formulation comprised 21.0% TAG, 77.8% DAG, 1.1% MAG, and 0.1% FFA. For fatty acids, the placebo oil comprised 3.9% palmitic acid (C16:0), 3.0% stearic acid (C18:0), 83.8% oleic acid (C18:1), 6.0% linoleic acid (C18:2), 0.3% alpha-linolenic acid (C18:3), and 3% other fatty acids. The ALA-DAG oil comprised 2.7% C16:0, 1.6% C18:0, 23.5% C18:1, 15.3% C18:2, 53.3% C18:3, and 3.6% other fatty acids. The ALA-DAG and the placebo oils could not be distinguished by appearance, taste, or odor. Test foods were provided to the subject after concealment by a person not involved in the study. Participants consumed the test oils, individually packaged in sachets weighing 2.5 g each, once daily with breakfast, lunch, or dinner for 8 weeks.

### Skin property measurements

Skin properties were measured twice, before and at 8 weeks after intake of the test oil. After washing the face, participants waited in a variable-environment room (temperature 20 ± 2 °C, humidity 40 ± 5%) for 20 min for acclimation. Afterward, TEWL was measured on the cheek using a Tewameter® TM300 (Courage + Khazaka Electronic GmbH, Koln, Germany), and stratum corneum water conductance was measured using a skin surface hygrometer (SKICON-200EX®; IBS Co., Hamamatsu, Japan). Skin color, brightness (L*), redness (a*) and yellowness (b*) were assessed by photographing the entire face using a hyperspectral imaging sensor (NH-7, Eba-Japan, Japan) and integrating a sphere lighting (CCS, Japan) system under normal temperature and humidity conditions and the D65 standard light source^46^. Total values for the forehead, both cheeks, eye area, and nose area were calculated from the spectral reflectance images.

### Assessment of skin and allergy symptoms

Before and at 4 and 8 weeks after intake of the test oils, participants’ subjective skin symptoms were assessed using the Japanese version of Skindex-16 (publisher; MPR K.K.)^47^. The 16 response options were presented on a 7-point scale, and as in the United States (US) version of the Skindex-16^48^, responses were converted to a linear scale of 100, ranging from 0 (not at all concerned) to 100 (always concerned). The mean of the 16-item scale was calculated, as well as the mean of the scale of the responses to the items belonging to “symptoms,” “emotions,” and “functioning. The original Japanese quality-of-life questionnaire (JRQLQ no. 1) was used to assess subjective allergy-like symptoms^49^.

### Blood analysis

Allergy tests were performed before and at 8 weeks after intake of the test oil. Serum total IgE levels and allergen-specific IgE levels (mite-specific IgE [d1] and Japanese cedar pollen-specific IgE [t17]) were quantified using the ImmunoCAP system (total IgE code no. 01813 [14–4509-01]; allergen-specific IgE code no. 30109; Thermo Fisher Scientific, Waltham, MA, USA) at LSI Medience Corporation (Tokyo, Japan). Sensitization was defined as IgE levels ≥ 0.35 U_A_/mL. Nine fatty acids, including palmitic acid, stearic acid, oleic acid, linoleic acid, dihomo-gamma-linolenic acid, arachidonic acid, ALA, eicosapentaenoic acid, and docosahexaenoic acid, were analyzed in blood before and at 8 weeks after intake of the test oils. Plasma was separated from the collected blood, and lipids were extracted according to the method of Folch et al.^50^. After adding chloroform/methanol to the plasma and stirring, distilled water was added, and the lipid fraction was obtained from the lower layer, which was then saponified to methyl esters. Hexane and saturated NaCl solution were then added, and the hexane layer was collected to make fatty acid methyl ester samples. The prepared samples were separated using a CP-Sil 88 capillary column (Chrompack, Middelburg, The Netherlands) as follows: 150 °C (5 min), 1 °C/min to 160 °C (5 min), 2 °C/min to 200 °C (10 min), 10 °C/min to 220 °C (5 min), and detected with a 6890N gas chromatograph equipped with dual flame-ionization detection systems (Agilent Technologies, CA, USA). The area of each fatty acid peak was divided by the sum of the areas of the nine fatty acid peaks to calculate the percentage composition of each fatty acid.

### Analysis of gene expression in skin surface lipids (SSL)

Sebum was collected from the entire face before and at 8 weeks after intake of the test oil using an oil-blotting film (3 M Japan, Tokyo, Japan). RNA extraction, library preparation, sequencing, and data preprocessing were performed as described previously^39–41,51^. In brief, SSL RNA was extracted using QIAzol (QIAGEN, Hilden, Germany) and purified using the Rneasy Mini Kit and QIAcube (QIAGEN). Sequencing libraries were prepared using the Ion AmpliSeq Transcriptome Human Gene Expression Kit (Thermo Fisher Scientific). Library quality was assessed using High-Sensitivity D1000 ScreenTape, and library quantity was determined with the Ion Library TaqMan™ Quantitation Kit (Thermo Fisher Scientific). Libraries were processed on the Ion Chef System (Thermo Fisher Scientific) for template preparation and chip loading, and RNA-Seq was performed using the Ion S5 XL System (Thermo Fisher Scientific).

### Statistical analysis

For skin properties and IgE levels, changes from baseline to after 8 weeks were compared between groups with the Wilcoxon rank-sum test. For the Skindex and JRQLQ scores, the data at baseline and at 4 and 8 weeks were analyzed using a repeated-measures analysis of variance adjusted for baseline, with group, time, and group-by-time interactions as fixed effects, and subjects as a random effect. The group effect and group-by-time interactions were evaluated using a generalized estimating equation (GEE). Least-squares means at week 4 and week 8 were also estimated and compared between groups. Correlation was assessed using Pearson’s correlation coefficient. Data with allergen-specific IgE antibody concentrations < 0.1 were treated as missing values. In the subgroup analysis, participants were divided into two groups: those were mite-specific IgE(+) at baseline and those who were mite-specific IgE(-). The results are presented as the means ± SD or means ± SE. *P*-values < 0.05 were considered statistically significant. For analyzing SSL gene expression, DEGs were identified using a likelihood ratio test with Benjamini-Hochberg’s FDR < 0.05. Gene ontology analysis of the DEGs was performed using the Database for Annotation, Visualization, and Integrated Discovery (DAVID v6.8)^52,53^. Statistical analyses and figure generation were conducted using IBM SPSS Statistics version 28 or 29 (IBM Corp., Armonk, NY, USA) and R version 4.4.3 (R Foundation for Statistical Computing, Vienna, Austria).

## Supplementary Information

Supplementary Information.

## Data Availability

The dataset from which the current study is not available to public for confidentiality reason, since the participants in this study agreed to use the data only for the analysis of present study when the study was informed and would not be shared with third parties, but is available from the corresponding author upon reasonable request.
