# Supplementary material for: Effects of diacylglycerol-enriched alpha-linolenic acid oil on skin properties in mild skin discomfort: a randomized, double-blind, placebo-controlled study
Source: Sci Rep. 2026 Jan 6;16:4785. doi: 10.1038/s41598-025-34887-3 (PMC12873180; doi:10.1038/s41598-025-34887-3)
Supplement: Supplementary file 1 — Supplementary Information. [file 41598_2025_34887_MOESM1_ESM.pdf]

# Supplementary information

**Title**

**Effects of diacylglycerol-enriched alpha-linolenic acid oil on skin properties in mild skin discomfort: a randomized, double-blind, placebo-controlled study.**

Authors
Satoko Fukagawa[1,2]*, Yoshie Shimotoyodome[1], Keimon Sayama[2,3], Aya Sasaki[1], Katsuyoshi Saito[1], Hiroki Fujita[3], Yuki Shimizu[1], Koichi Misawa[1], Shinichiro Saito[1], Junko Ishikawa[2,3], Noriyasu Ota[3], Takehiko Yokomizo[2], Masanobu Hibi[1]

Affiliations
[1] Human Health Care Products Research, Kao Corporation, Bunka 2-1-3, Sumida, Tokyo 131-8501, Japan
[2] Department of Biochemistry, Juntendo University Graduate School of Medicine, Hongo 2-1-1, Bunkyo, Tokyo, 113-8421, Japan
[3] Biological Science Research, Kao Corporation, Akabane 2606, Ichikai-machi, Haga-gun, Tochigi, 321-3497, Japan.

* Corresponding Author
Satoko Fukagawa, Human Health Care Products Research, Kao Corporation, Bunka 2-1-3, Sumida, Tokyo 131-8501, Japan, fukagawa.satoko@kao.com

**Supplementary table 1:** Fatty acids in the blood

| Parameter | Unit | Group | n | 0 w | | | 8 w | | | |
|---|---|---|---|---|---|---|---|---|---|---|
| **Palmitic acid (16:0)** | % | Placebo | 29 | **22.19** | ± | **0.38** | **24.08** | ± | **0.37** | ## |
| | | ALA-DAG | 29 | **22.57** | ± | **0.43** | **24.64** | ± | **0.37** | ### |
| **Stearic acid (18:0)** | % | Placebo | 29 | **8.74** | ± | **0.17** | **9.14** | ± | **0.16** | |
| | | ALA-DAG | 29 | **8.82** | ± | **0.16** | **9.07** | ± | **0.18** | |
| **Oleic acid (18:1 n-9)** | % | Placebo | 29 | **21.06** | ± | **0.57** | **20.26** | ± | **0.50** | |
| | | ALA-DAG | 29 | **21.43** | ± | **0.64** | **20.93** | ± | **0.50** | |
| **Linoleic acid (18:2 n-6)** | % | Placebo | 29 | **32.46** | ± | **0.76** | **31.04** | ± | **0.57** | |
| | | ALA-DAG | 29 | **31.50** | ± | **0.89** | **30.39** | ± | **0.56** | |
| **Dihomo-gamma-linolenic acid (20:3 n-6)** | % | Placebo | 29 | **1.48** | ± | **0.07** | **1.30** | ± | **0.06** | # |
| | | ALA-DAG | 29 | **1.46** | ± | **0.06** | **1.27** | ± | **0.07** | # |
| **Arachidonic acid (20:4 n-6)** | % | Placebo | 29 | **8.09** | ± | **0.30** | **8.07** | ± | **0.21** | |
| | | ALA-DAG | 29 | **7.70** | ± | **0.27** | **7.56** | ± | **0.23** | |
| **alpha-Linolenic acid (18:3 n-3)** | % | Placebo | 29 | **0.71** | ± | **0.03** | **0.76** | ± | **0.03** | |
| | | ALA-DAG | 29 | **0.71** | ± | **0.04** | **0.87** | ± | **0.05** | # |
| **Eicosapentaenoic acid (20:5 n-3)** | % | Placebo | 29 | **1.35** | ± | **0.17** | **1.58** | ± | **0.26** | |
| | | ALA-DAG | 29 | **1.63** | ± | **0.19** | **1.36** | ± | **0.14** | |
| **Docosahexaenoic acid (22:6 n-3)** | % | Placebo | 29 | **3.92** | ± | **0.22** | **3.77** | ± | **0.23** | |
| | | ALA-DAG | 29 | **4.18** | ± | **0.20** | **3.91** | ± | **0.19** | |

Fatty acids levels (% of total fatty acids) in the blood at baseline and after the 8-week intervention. Data are expressed as mean ± SE. (Placebo group, n = 29; ALA-DAG group, n = 29). Values at the initial measurement and after 8 weeks of continuous intake were assessed using the paired *t*-test.
# $P < 0.05$, ## $P < 0.01$, ### $P < 0.001$.
Abbreviations; ALA-DAG, diacylglycerol-enriched alpha-linolenic acid oil; SE, standard error.

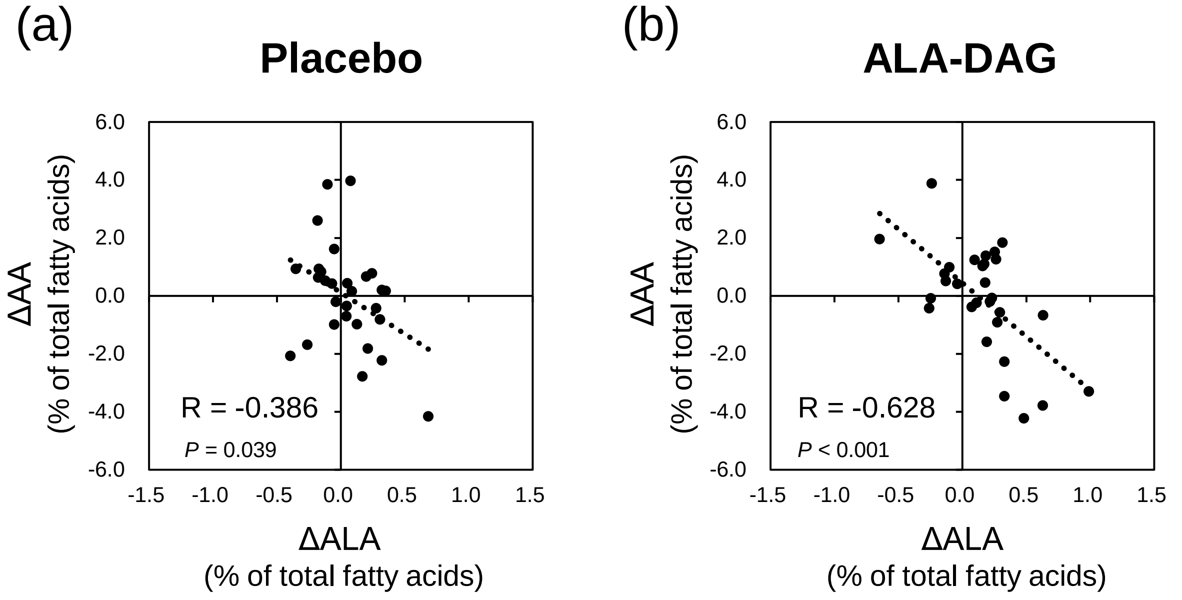


**Supplementary figure 1**: Correlations between changes in ALA and arachidonic acid in the blood after 8 weeks of intervention in the placebo group (a) and the ALA-DAG group (b). The correlation analysis was performed by calculating Pearson's correlation coefficient.

Abbreviations; AA, arachidonic acid; ALA, alpha-linolenic acid; ALA-DAG, diacylglycerol-enriched alpha-linolenic acid oil.

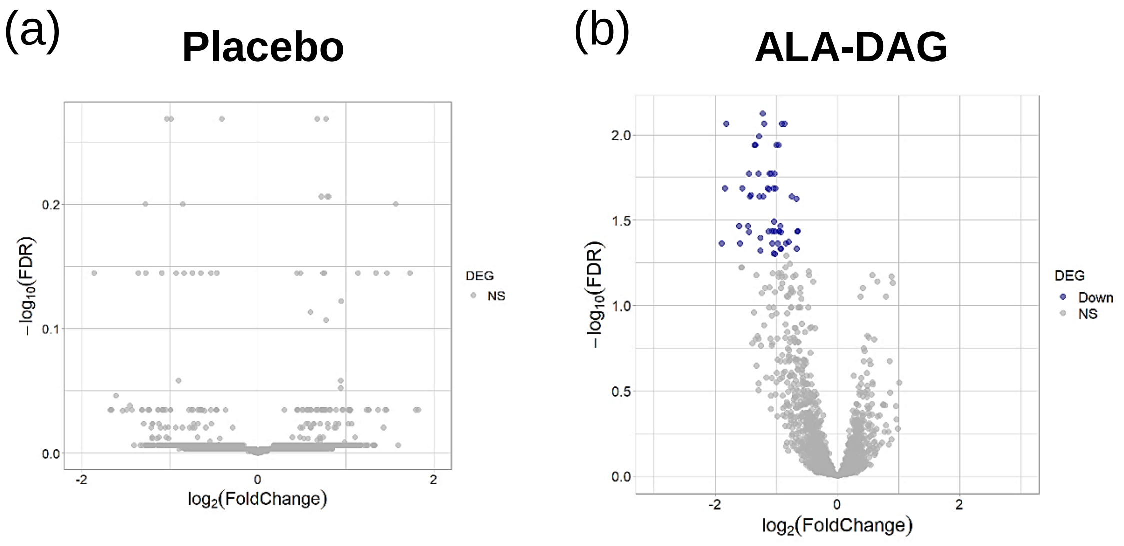


**Supplementary figure 2**: Volcano plot of differentially expressed genes in skin surface lipids in the placebo groups (a) and the ALA-DAG groups (b). Downregulated genes after 8 weeks of intervention are indicated by blue spots. Benjamini and Hochberg's FDR < 0.05 as a threshold.

Abbreviations; ALA-DAG, diacylglycerol-enriched alpha-linolenic acid oil; DEG, differentially expressed gene; FDR, false discovery rate; NS, not significant.

**Supplementary table 2:** Gene ontology analysis of downregulated mRNAs in skin surface lipids from the mite -specific IgE(+) ALA-DAG group

| GO term | Gene Count | FDR |
|---|---|---|
| **Response to external stimulus** | 30 | 4.7E-09 |
| **Response to biotic stimulus** | 22 | 4.7E-09 |
| **Immune system process** | 31 | 6.9E-09 |
| **Response to other organism** | 21 | 8.8E-09 |
| **Response to external biotic stimulus** | 21 | 8.8E-09 |
| **Response to cytokine** | 19 | 2.3E-08 |
| **Immune response** | 25 | 5.0E-08 |
| **Defense response** | 24 | 5.8E-08 |
| **Response to organic substance** | 30 | 5.8E-08 |
| **Inflammatory response** | 17 | 6.1E-08 |
| **Neutrophil chemotaxis** | 9 | 8.3E-08 |
| **Leukocyte migration** | 13 | 1.2E-07 |
| **Cellular response to organic substance** | 26 | 2.7E-07 |
| **Neutrophil migration** | 9 | 3.7E-07 |
| **Granulocyte chemotaxis** | 9 | 4.1E-07 |
| **Response to lipopolysaccharide** | 12 | 4.3E-07 |
| **Response to bacterium** | 16 | 5.4E-07 |
| **Cell surface receptor signaling pathway** | 27 | 5.8E-07 |
| **Cytokine production** | 16 | 5.8E-07 |
| **Cellular response to chemical stimulus** | 28 | 5.8E-07 |
| **Response to molecule of bacterial origin** | 12 | 6.2E-07 |
| **Regulation of immune system process** | 21 | 6.7E-07 |
| **Cellular response to cytokine stimulus** | 16 | 9.9E-07 |
| **Granulocyte migration** | 9 | 1.0E-06 |
| **Response to interferon-gamma** | 9 | 1.1E-06 |
| **Positive regulation of cytokine production** | 13 | 1.2E-06 |
| **Innate immune response** | 17 | 1.2E-06 |
| **Regulation of response to stimulus** | 32 | 1.2E-06 |
| **Cell chemotaxis** | 11 | 1.3E-06 |
| **Leukocyte chemotaxis** | 10 | 1.5E-06 |
| **Signal transduction** | 37 | 2.5E-06 |
| **Response to chemical** | 32 | 4.0E-06 |
| **Positive regulation of response to stimulus** | 24 | 4.2E-06 |
| **Positive regulation of response to external stimulus** | 11 | 4.4E-06 |
| **Positive regulation of immune system process** | 17 | 4.4E-06 |
| **Regulation of cytokine production** | 15 | 4.6E-06 |
| **Signaling** | 38 | 4.6E-06 |
| **Cellular response to interferon-gamma** | 8 | 5.2E-06 |
| **Cell communication** | 38 | 5.3E-06 |
| **Acute inflammatory response** | 8 | 7.9E-06 |
| **Response to stress** | 30 | 8.3E-06 |
| **Regulation of response to external stimulus** | 15 | 1.1E-05 |
| **Cytokine-mediated signaling pathway** | 12 | 1.2E-05 |
| **Single organism signaling** | 37 | 1.4E-05 |
| **Myeloid leukocyte migration** | 9 | 1.5E-05 |
| **Response to oxygen-containing compound** | 19 | 1.6E-05 |
| **Regulation of defense response** | 14 | 2.0E-05 |
| **Cell migration** | 18 | 2.0E-05 |
| **Regulation of immune response** | 15 | 2.6E-05 |
| **Multi-organism process** | 23 | 2.6E-05 |
| **Positive regulation of defense response** | 11 | 3.0E-05 |
| **Cell motility** | 19 | 3.2E-05 |
| **Localization of cell** | 19 | 3.2E-05 |
| **Response to tumor necrosis factor** | 9 | 3.5E-05 |
| **Locomotion** | 20 | 3.5E-05 |
| **Chemotaxis** | 12 | 4.2E-05 |
| **Taxis** | 12 | 4.4E-05 |
| **Immune effector process** | 14 | 4.4E-05 |
| **Cellular response to stimulus** | 39 | 5.5E-05 |
| **Response to lipid** | 14 | 5.6E-05 |
| **Response to stimulus** | 43 | 6.6E-05 |
| **Defense response to bacterium** | 10 | 8.2E-05 |
| **Regulation of innate immune response** | 10 | 8.5E-05 |

Abbreviations; FDR, false discovery rate.
